# Gleanings from Our Exchanges

**Published:** 1872-03-01

**Authors:** 


### Gleanings from Our Exchanges.

### A CASE OF HYDROCELE OF THE ROUND LIGAMENT MISTAKEN FOR AND OPERATED UPON AS A STRANGULATED HERNIA, WITH REMARKS.

BY CHAS. A. HART, M.D., NEW YORK.

*From Monograph, published by D. Appleton & Co.*

March 3, 1867.—Mrs. P., aged about forty-five years, was thought by her family attendant, Dr. B., to have a strangulated femoral hernia; he called upon Dr. J. M. C. to operate for him, who, after having visited the patient, called upon Dr. R. Nelson to mention the case, and consult with him, giving him the following imperfect history: "She had had more than one child: she thinks that the tumor in her right groin appeared about fifteen years ago, after straining in labor; she had never been able to reduce it, nor had it ever given her pain or inconvenience." What her present illness may be, Dr. C. does not know; he has seen her only once, having been called by her family doctor to operate for *strangulated femoral hernia*, which is all he has to do in the case. Dr. C. suspects chronic incarceration, and at present strangulation, *because the patient has vomited once.* He has tried taxis ineffectually. He says, "It is surprising how soft the tumor is, and remarkably thin the parietes are."

The patient was very much averse to any surgical interference, but, having consented to an operation at the urgent request of her husband and medical attendant, at 8 o'clock was brought under the influence of chloroform, while in bed; and, when fully anæsthetized, carried into the front parlor (where several assistants were waiting), and placed upon a table for operation. The tumor was now exposed, which was found to be larger than a hen's egg, flattened, situated *above Poupart's ligament, and in the direction of the inguinal canal.* Dr. C. made a slight and ineffectual attempt at taxis, which served only to drive the contents of the tumor in a contrary direction, to one or the other side, or upward, which as soon as pressure was discontinued, returned to its former size, site, and appearance.

Dr. N. was requested to examine the tumor, and did so, recognizing at once its nature, for he immediately placed his hand above the tumor to exclude lateral light, and requested me to hold a candle close to it, which I did, when the *diaphanous character of hydrocele* became strikingly apparent. *The parietes of the tumor were not tense, but soft.* There was no opportunity afforded for advice or caution, as Dr. C. immediately commenced his operation by making an incision through the skin *transversely across the abdomen*, at least *an inch and a half above Poupart's ligament*, about four inches long, and another incision bisecting the first vertically, about three inches in length; the upper line of this cut lay on the abdomen, the lower one across the tumor as far as its lower border, bringing into view a smooth, bluish, and translucent sac. The four corners of the crucial cuts were dissected up, and the whole tumor exposed, which could now no more be reduced than before. A prolonged attempt was now made to separate an expectant fascia, by repeated scratching and division of thin laminæ of cellular tissue, raised by thrusting a director under the leaves, one after another, without meeting any, until the parietes of the tumor became so thin that the contained fluid began to ooze out. (The operator, thinking the blue tumor was intestine, was surprised that the sac had no fascia.) Dr. N. repeatedly advised, "*Cut into it, it is not intestine you have here*," when Dr. C. thrust an exploring needle into it, on the withdrawal of which about three ounces of a clear, limpid fluid spurted out, arch-form, resembling hydrocelic fluid; at this stage the supposed hernial tumor quite disappeared. The sac which had contained the fluid was now freely laid open in search of intestine, or omentum, but neither was found; but a long, thick, fatty mass lay in the *canal of the round ligament*, free in front, but adherent behind. This was in *no way inflamed or discolored;* it was said to be incarcerated omentum, but attempts to either reduce it or pull it down were equally unavailing. After considerable manipulation the lower end became detached from its adhesions to the canal, and by pushing and tearing was separated as far up as the internal inguinal ring, when an ineffectual attempt was again made to return it within the abdomen. This failing, it was decided to cut it off; before this could be carried into execution, Dr. N. managed to press his fingers through the elongated meshes of which it consisted, and in this way severed it into four or five strips, three of which, supposed by the operator to be omentum, were cut off, from the ends of which a few drops of blood oozed, furnished by a couple of arterioles the size of fine hairs: these were ligated. The determination being to make the case one of hernia, the finger was worked in the direction of the canal into the abdomen, and the remaining mass thrust in, and the wound closed by wire sutures, compresses, etc.

The little blood that oozed came from a fleshy fibre of the hypertrophied fibres of an analogous cremaster muscle. The fatty structure had not the slightest resemblance to omentum, but resembled in some degree a spermatic cord, and was composed of granular fat.

The after-history of the case (except that recovery had taken place), I remained in ignorance of until October 26, 1870, when, having an opportunity of seeing the patient, she furnished me as clearly as she could remember the history of her complaint from the time of discovery, about eighteen years ago, when she first noticed the tumor while bathing, about two or three months after a not severe confinement. The tumor was then hardly the size of a filbert, and unattended with pain; she made repeated efforts to reduce it, supposing it to be a hernia, but never succeeded, nor has it ever disappeared during the recumbent posture. The tumor, without causing either pain or inconvenience, steadily but slowly increased until it attained the size it was at the time of operation in 1867; following which she kept her bed for six weeks, though the wound was closed at the end of three. A short time after, being about again, she noticed a commencing return of the tumor, which at the present time has attained about the same size as the original one, and, like it, of gradual growth and free from pain. Her former experience not being of a pleasing kind, she has adopted the precaution of wearing a truss (being still under the impression that the tumor is hernial), but with the effect of merely flattening the tumor, as it has never been reduced.

I regret exceedingly that the lady's extreme repugnance to an examination could not be overcome, as more information might have been gained. The pathological conditions found in this case can, I presume, be considered as typical of this extremely rare form of disease; of which I know of but two other instances which came under the observation of Dr. R. Nelson, both of which possessed the same physical characteristics of *translucency, slow growth, non-reducible, and free from pain;* no operation was performed in either case.

The literature upon this form of disease is extremely scanty, it being mentioned, as far as I am aware, only in the older editions of Ætius, Pare, Scarpa, Meckel, and Poland. That the disease occurs more frequently than is generally supposed, there can be but little doubt; but, not being mentioned or classified in the modern medical works, it is confounded with other diseases, and not recognized in its true nature.

---

Respiratory Murmurs.—At a stated meeting of the New York Academy of Medicine, Dr. James R. Leaming read a paper on "Respiratory Murmurs," of which we present an abstract. He starts out with the purpose of proving the composite character of the respiratory murmur. Referring to the anatomy of the tissue of the lungs and bronchial system, he calls particular attention to the circulations in the bronchial system; the bronchial arteries are attended by venæ comtes only so far as the bronchial tubes extend, and not into the true pulmonary structure, consequently the blood which goes to nourish this structure does not return to the heart as venous blood but is ærated in the air vesicles and returned by the pulmonary vein as arterial blood to the right side of the heart. Occupation of the air-sacs by tubercle interferes with the circulation of the nutrient artery, and blood is thrown back upon the bronchial artery, resulting in bronchonagia—a conservative act, for, like the application of leeches, it sets the absorbents actively at work to remove the cause. In this way cases of early phthisis are self-cured, or, at all events, ameliorated, and the physician is guided in his treatment. After some further comments on this manner of circulation the writer goes on to speak of the residual air in the lungs, and that it has no more motion than the water in the bottom of the ocean—the air in the air-cells is purified by the laws of the diffusion of gases. Hence, any theory of vesicular murmur or crepitant rale which ignores the existence of this residual air must of necessity be incompetent. The respiratory murmur consists of two sounds: the first, the motion of the tidal air in the bronchæ; the second, due to the contraction and dilatation of the muscular (?) fibres of the lung substances; the first is intermittent, the second continuous, and may be heard best when the breath is held. This, the true respiratory murmur, only begins to be developed after eight years of age, becomes recognizable at twelve, and is only fully developed at maturity. *The Broncho-Respiratory Murmur.*—The sound produced by the tidal air may be heard by forcing the respiration, thereby increasing the velocity of the air in the bronchial tubes and the loudness of the sound. It is heard most perfectly, however, in children before the true respiratory murmur begins.

It is said that the vapor of a few drops of nitrite of amyl, cautiously inhaled, will relieve the dyspnœa of an attack of spasmodic asthma almost instantly. Dr. H. C. Wood, jr., New Remedies, Jan., 1872, says that he has used it with success in one case of aninga pectoris dependant on valvular disease of the heart, when all other remedies had failed.

Dr. Charles Bell Taylor, (*London Lancet*, Feb., 1872,) describes a new way of extracting cataract. The instruments he employs are a pair of toothed-forceps, an eye speculum, and two knives a line in width, one sharp pointed, the other blunt pointed, and both bent at an angle like an iridectomy knife. The lids are to be separated with the speculum, the eyeball turned downwards with an ordinary pair of forceps, and fixed in a favorable position by the application of the toothed-forceps at about the junction of the middle with the upper third of the rim of the cornea. The pointed knife is then entered at the sclero corneal junction near the attachment of the forceps; it is pushed well into the anterior chamber, and, with a gentle, sawing motion, carried along the upper border until about one-third of the cornea has been divided. The eyeball is to be fixed with a pair of ordinary forceps—the capsule of the lens is to be made tense and is then to be carefully divided with Von Graafi's cystitome. The capsule is divided at this stage of the operation for the sake of avoiding the obscuration of the view of the operation by the effusion of blood into the anterior chamber, as would be the case if it was postponed until after the next step. After the tearing of the capsule a small portion of the upper part of the iris is to be seized, and a small portion of the periphery excised, leaving the pupillary margin intact. The lens is then extracted in the ordinary way, gliding behind the pupil without stretching the sphincter. By this method of operating, he claims to have obtained excellent results, far surpassing that from other methods, with very small percentage of bad results.

Pitting in Small-pox.—In regard to the very numerous methods for preventing small-pox pitting, the truth appears to be that none is so thoroughly effective and to be relied on, but that anything which excludes thoroughly the light and air will have a certain amount of influence in lessening the chances of severe disfigurements.

Dr. Rendle, in the London *Practitioner*, of October, writes as follows: — He says, "I have now two cases convalescent from small-pox, in which I applied cotton wool to protect the face. The disease in each case was of the distinct form. One of the two, a girl, aged fifteen years, had an abundant eruption, which, in the unprotected parts of the body, went through the usual consecutive changes. In both cases the parts covered with the wool are left without a vestige of marks. The mode of application is as follows: On the first appearance of the eruption, patches of skin, about an inch square, were washed over with collodion, and immediately covered over with a thin uniform layer of fine wool; the wool readily adheres if applied before the ether of the collodion evaporates. When the whole of the face, etc., was thus covered, the wool was brushed over with a solution of starch or gum. The starch or gum was occasionally reapplied to the edges of the wool, to prevent any shifting by the movements of the face. This covering was kept on until the dry crust fell off the other parts of the body."

ALCOHOL AS A MEDICINE. — Dr. Forbes Winslow, the well-known writer on diseases of the brain and mind, has sent a letter to the London *Times*, stating that during the last twenty years he has seen numerous cases, more particularly among women, of an insane craving for alcohol, which could be traced to the injudicious use of stimulants given, in the first instance, medicinally. The unwise and prolonged continuance of the use of alcohol after the physician has retired from the treatment of the case, Dr. Forbes Winslow says, causes spirits to become a necessity of life, inducing habits of tippling and confirmed drunkenness, and eventually developing severe diseases of the brain and mind, and frightful disorders of the nervous system. Dr. Forbes Winslow speaks in the strongest terms of condemnation of the stimulating theory as applied to the treatment of acute inflammatory affections. He says that the habit of stimulation may be established by the occasional sipping of spirits of wine, cologne water, spirits of chloroform, spirits of ammonia or any of the medicinal tinctures, as well as by drinking brandy, whisky or wine. Such opinions are not yet indorsed by the profession at large with us.

EPILEPSY IN A GOUTY SUBJECT CURED BY COLCHICUM.—Dr. Rounel was called to see M. P., a bailiff, by his son, who said his father was dying in an epileptic fit. By the time the doctor arrived the fit had passed, and the man was able to tell him that he had suffered from epilepsy for ten years, and had consulted many noted physicians; but in spite of the most faithful trial of their treatments, the paroxysms were constantly becoming more frequent and severe. The various prescriptions (copies of which had been kept) comprised almost all the recognized anti-epileptic drugs. On questioning it was found that the patient had formerly been a martyr to gout—that a severe gouty attack had immediately preceded the first epileptic paroxysm—since which he had been free from his disorder. He was at once put upon the steady use of colchicum, and after a short time was freed from his epilepsy.—*Revue de Therapeut. Medico-Chirurg.*

INCREASE OF INTEMPERANCE IN FRANCE.—At the last session of the Academy of Medicine Drs. Magnan and Bouchereau gave the result of their research among the sick who entered the Sainte Anne Hospital during March, April and May, 1871, either because of tremens or paralysis. They endeavor to show that it is not alone by the larger number that the "alcoholists" of 1870–1 are distinguished from those of former years, but by the pronounced character of the intoxication. The most salient fact is the enormous proportion of persons struck down by general paralysis. Alcohol, during the month of May, 1871, furnished more than half the total contingent of insane to the asylums.

PHYSIOLOGICAL ACTION OF PUTTY.—A correspondent of the Philadelphia *Times* relates a case of a child who was troubled during October with constipation and acute strangury, making frequent attempts to void water, attended with much pain and screaming. There was no fever, the pulse was natural, tongue a little red, and the gums swollen from the imminent eruption of several teeth. The urine was "scanty, rather high-colored, and contained no albumen or casts, but a considerable number of very small oil-globules." These symptoms occurred at several different times, and were discovered, on careful investigation, to have been occasioned by handling and eating putty.

MEDICAL EDUCATION OF WOMEN.—This subject has again been brought to the attention of the authorities of the Medical University of Edinburgh, and permission for females to attend lectures and receive degrees under the same conditions as males, has been refused. The governors of the University, however, are willing that medical instruction should be imparted to women in strictly separate classes, provided the female students would be satisfied in receiving, after examination, certificates of proficiency in medicine instead of University degrees. Such certificates, it is stated, are granted by the London University.

THE MOTHER OF TWO PHYSICIANS.—Mrs. Southworth, the novelist, is thus chattered about: "Tallish in figure, with full forehead, well balanced head, thoughtful gray eyes, and a face denoting intellect of the deliberate, reasoning kind, she seems likelier to be a writer of the Martineau order than of the imaginative style. She has two children: Dr. Richard J. Southworth, a much esteemed physician of Georgetown, and Charlotte Emma Lawrence, the wife of Dr. James V. Lawrence, and officer in the United States Army."

TREATMENT OF GRANULAR OPHTHALMIA BY QUININE.—C. Bader, Ophthalmic Assistant Surgeon to Guy's Hospital, reports in the *Lancet*, for Feb., 1872, several cases of granular ophthalmia treated with powdered bi-sulphate of quinine. As much as would be held on the point of a penknife was dusted over the granulations twice a day. He reports excellent results, especially in cases of long standing, in which there was much pannus.

Sulphur versus Small-pox. — The chief physician of Iceland claims to have smoked out the small-pox, lately imported to that country from France, by means of sulphur, with the aid of sulphurous acid and water drank by the patients. The disease disappeared, and no new cases had occurred for thirty days.—*Canada Lancet, Feb.*, 1872.

Antidote to Liquor Poisoning.—The physician in charge of the drunkard's department of Blackwell's Island, says that milk or eggs are antidotes for liquor poisons, and those who must drink liquor should mix milk or eggs with their " poison," which will take effect on those articles instead of the toper's stomach.

Tetanus has been cured in France, in a number of cases, by extremely hot air baths, followed by hypodermic injections of morphia.

